# Children’s Social Behaviors: Developmental Mechanisms and Implications

**DOI:** 10.3390/bs14030230

**Published:** 2024-03-12

**Authors:** Xuechen Ding, Wan Ding

**Affiliations:** 1 School of Psychology, Shanghai Normal University, Shanghai 200234, China; 2 Laboratory for Educational Big Data and Policymaking, Ministry of Education, Shanghai 200234, China; 3 Parent Education Research Center, School of Psychology, Zhejiang Normal University, Jinhua 321004, China

**Keywords:** social behavior, parent, peer, adjustment, neuroscience

## Abstract

During the socialization process in family and school contexts, children display a wide variety of social behaviors with parents and peers. Yet the developmental trajectory, the predictors and outcomes, and the neural basis of those social behaviors are largely under-investigated. To address these problems, we invited experts in the field to submit their latest findings to tell this story. The current Special Issue is a collection of papers highlighting the complexity for various social behaviors, with a focus on the complex mechanisms that link social behaviors to child socio-emotional adjustment and mediating/moderating factors among the associations. Thirteen papers illustrate empirical work in the field, two papers present new methodological concerns, and one paper that provides a comprehensive review of the literature.

Normative development of children’s social, emotional, and school adjustment stems from sources including both family and peer groups. Parent–child and peer interactions have unique and significant implications for children, and these social relationships serve as a foundation for feelings of security and belonging. During the socialization process in family and school contexts, children display a wide variety of social behaviors. However, several issues require further exploration. For example, what are the different causal relations that might underlie children’s social behaviors? What is the trajectory of these social behaviors at different developmental stages? Could we find the neural basis for these social behaviors via advanced techniques? In light of these premises, this Special Issue aims to advance the literature on the development trajectory of children’s social behaviors and their cognitive neural mechanisms. Sixteen papers in this Special Issue elucidate a broad range of social behaviors and provide us two important concerns.

## 1. Behavior Type Concerns

The authors in this Special Issue focus on a variety number of social behaviors, including social withdrawal, prosocial behavior, parent-child interaction, and problem behavior. 

Three studies revealed the implication of social withdrawal. Overall, most of the withdrawn subtypes turn out to be risk factors in the development of child psychological well-being. Bowker et al. (contribution 1) examined the associations between anxious-withdrawal and sleeping difficulties and found that social withdrawal longitudinally predicted later trouble sleeping, especially for excluded and victimized adolescents. Sette et al. (contribution 2) used a person-oriented approach to create shy, unsociable, avoidant, and non-withdrawn groups, and found that unsociable group appeared to be the most well-adjustment group whereas shy and avoidant group reported higher level of internalizing problems. Zhu et al. (contribution 3) examined the associations between shyness and indices of socio-emotional adjustment and found that higher levels of screen time exacerbated the associations.

Two studies focus on prosocial behavior, another common social behavior in childhood and adolescence. Li et al. (contribution 4) examined the associations between prosocial behavior and psychological maladjustment, and found that peer preference and self-perceived social competence played as a serial indirect pathway among these associations. Liu et al. (contribution 5) reported that psychological suzhi mediated the effects of early emotional experiences on prosocial behavior and subjective socioeconomic status moderated the mediation model. 

Three studies showed some interesting findings of parent-child interaction. Specifically, all these parent-child interaction variables seem to be mediators in the development of child adjustment outcomes. Wang et al. (contribution 6) found that stress from context and parenting would lead to more maternal psychologically controlling in parenting, and in turn lead to less socio-emotional difficulties. Geng et al. (contribution 7) examined the associations between electronic media use and internalizing problems, and further found that parent–child conflict was a salient mediator among the associations. He et al. (contribution 8) examined the mechanism in predicting ODD child emotional problems through system, dyadic, and individual levels, and found that parent-child attachment played as a mediator role. 

Two studies examined the relevant predictors for the development of problem behavior. Yang et al. (contribution 9) examined the developmental trajectory of aggressive behavior, and the predictors on its intercept and slope from parent and child self (i.e., corporal punishment, psychological aggression, and self-esteem). Mo et al. (contribution 10) examined the associations between family SES and later problem behavior via a longitudinal design, with sense of coherence as mediator and maternal warmth as moderator.

## 2. Methodological Concerns

The papers in this Special Issue also illustrate some methodological concerns, particularly regarding sample selection, research design, and analytic strategy. One highlight is the broad age range from early childhood (contribution 3, contribution 6, contribution 7), middle childhood (contribution 8, contribution 10, contribution 11), adolescence (contribution 1, contribution 4, contribution 5, contribution 9, contribution 12), and emerging adulthood (contribution 2), and participants from different regions such as North America (contribution 1, contribution 6, contribution 11), Europe (contribution 2), and China (contribution 3, contribution 4, 5, contribution 7, contribution 8, contribution 9, contribution 10, contribution 12). The coverage of wide developmental stages and diverse cultural backgrounds make the conclusions in this Special Issue more generalizable to different context.

The second highlight is the inclusion of both cross-sectional and longitudinal design, and data collection from multiple informants. Five studies in the Special Issue used longitudinal design with time point from three months to two years (contribution 1, contribution 2, contribution 9, contribution 10, contribution 11). In addition, it should be noted that five studies collected their data from multiple informants rather than sole self-reports. Sources such as peer nominations (contribution 4), parent reports (contribution 1, contribution 6, contribution 7), and teacher-ratings (contribution 3, contribution 8, contribution 10) are all included in the Special Issue and these make the conclusions more reliable and robust. 

The third highlight is the inclusion of advanced technique and statistical method. Aside from traditional regression analysis, most of the papers in this Special Issue used advanced statistical strategy, such as Structural Equation Modeling (contribution 1, contribution 3, contribution 4, contribution 5, contribution 6, contribution 7, contribution 8, contribution 10), Latent Growth Modeling (contribution 9), and Latent Profile Analysis (contribution 2). Moreover, Chow et al. (contribution 11) collected neural data via EEG technique and found that frontal EEG alpha power moderated the association between shyness and anxiety. This study indicated the important function of cortical activity in social interaction for individual with different social motivations. Jiang et al. (contribution 12) conducted a machine learning technique to develop a shorter measurement (i.e., Buss–Warren Aggression Questionnaire) to increase the efficiency of assessing aggressive behavior, which could reduce the frequency of insufficient effort response and decrease the implementation costs.

## 3. Future Directions in Social Behavior Research

One suggestion for future research is the direct comparison for different age groups. The developmental mechanisms of children’s social behaviors may vary from early childhood to emerging adulthood because of their neurological, physiological, socio-cognitive, and emotional characteristics [1,2]. Future research would benefit from directly comparing the influence of interpersonal interactions on social behavior by collecting data from multiple age groups. In fact, some studies indicated that while parent-child interaction was related to children’s prosocial behaviors in early childhood, adolescents’ emotion in peer and teacher interactions may be more critical in predicting prosocial behaviors [3,4]. In addition, some researchers found the age differences in neural mechanism of prosocial behavior. For example, the neural activation in the posterior superior temporal sulcus (pSTS), temporal pole, inferior frontal gyrus (IFG), and cuneus in early adolescence are peaked compared with childhood and late adolescence, showing an inverted U-shaped curve [5].

The second suggestion is the use of more neuroscience techniques, such as fNIRS or fMRI-based hyperscanning approach. Different from employing traditional psychological methods such as questionnaires and behavioral experiments, the new techniques would be helpful to incorporate research approaches from social cognitive neuroscience and psychophysiology to explore the neural and physiological mechanisms underlying the development of children’s social behaviors [6]. It has been proposed that neural synchrony can enhance mutual understanding between children and others (parents, peers, or teachers) [7,8]. Some studies have already found the evidence that neural synchrony serves as a crucial neural mechanism underlying prosocial and cooperative behavior [9,10]. Indeed, the use of more neuroimaging techniques, such as hyperscanning, can capture more ecologically valid processes and mechanisms underlying the development of children’s social behaviors. 

The third suggestion is the use of qualitative method. In the current Special Issue, most of the papers were conducted via quantitative method, which may lead to more generalized findings [11]. However, it is difficult to reveal the developmental mechanism underlying social interaction in real life, as well as the details of psychological needs behind children and adolescents’ social behaviors. In future, more qualitative research should be encouraged, including behavior coding-based observation and semi-structured interviews [12,13], to deepening the developmental processes and mechanisms underlying the real and simulated interpersonal interactions.

## References

[1] Van der Meulen M., Dobbelaar S., van Drunen L., Heunis S., van IJzendoorn M.H., Blankenstein N.E., Crone E.A. (2023). Transitioning from childhood into adolescence: A comprehensive longitudinal behavioral and neuroimaging study on prosocial behavior and social inclusion. NeuroImage.

[2] McCormick E.M., van Hoorn J., Cohen J.R., Telzer E.H. (2018). Functional connectivity in the social brain across childhood and adolescence. Soc. Cogn. Affect. Neurosci..

[3] Chen W., Li X., Huebner E.S., Tian L. (2022). Parent-child cohesion, loneliness, and prosocial behavior: Longitudinal relations in children. J. Soc. Pers. Relatsh..

[4] Yang P.J., McGinley M. (2023). The associated effects of parent, peer and teacher attachment and self-regulation on prosocial behaviors: A person-and variable-centered investigation. J. Soc. Pers. Relat..

[5] Do K.T., McCormick E.M., Telzer E.H. (2019). The neural development of prosocial behavior from childhood to adolescence. Soc. Cogn. Affect. Neurosci..

[6] Reindl V., Wass S., Leong V., Scharke W., Wistuba S., Wirth C.L., Konrad K., Gerloff C. (2022). Multimodal hyperscanning reveals that synchrony of body and mind are distinct in mothe-child dyads. NeuroImage.

[7] Bi X., Cui H., Ma Y. (2023). Hyperscanning studies on interbrain synchrony and child development: A narrative review. Neuroscience.

[8] Ratliff E.L., Kerr K.L., Cosgrove K.T., Simmons W.K., Morris A.S. (2022). The role of neurobiological bases of dyadic emotion regulation in the development of psychopathology: Cross-brain associations between parents and children. Clin. Child. Fam. Psychol. Rev..

[9] Ishihara T., Hashimoto S., Tamba N., Hyodo K., Matsuda T., Takagishi H. (2023). Uncovering the links between physical activity and prosocial behaviour: A functional near-infrared spectroscopy hyperscanning study on brain connectivity and synchrony. bioRxiv.

[10] Koul A., Ahmar D., Iannetti G.D., Novembre G. (2023). Spontaneous dyadic behaviour predicts the emergence of interpersonal neural synchrony. NeuroImage.

[11] Gough B., Lyons A. (2015). The future of qualitative research in psychology: Accentuating the positive. Integr. Psychol. Behav. Sci..

[12] Başer Baykal N., Erden Çınar S. (2023). Understanding early maladaptive schemas formation with traumatic experiences in childhood: A qualitative study. J. Aggress. Maltreatment Trauma.

[13] Jiwon H., Younchul C. (2023). Young children’s metacognition expressed during coding play. Asia-Pac. J. Res. Early Child. Educ..

